# *Campylobacter jejuni* Colonization in Wild Birds: Results from an Infection Experiment

**DOI:** 10.1371/journal.pone.0009082

**Published:** 2010-02-05

**Authors:** Jonas Waldenström, Diana Axelsson-Olsson, Björn Olsen, Dennis Hasselquist, Petra Griekspoor, Lena Jansson, Susann Teneberg, Lovisa Svensson, Patrik Ellström

**Affiliations:** 1 Section for Zoonotic Ecology and Epidemiology, Linnaeus University, Kalmar, Sweden; 2 Department of Animal Ecology, Lund University, Lund, Sweden; 3 Department of Infectious Diseases, Uppsala University, Uppsala, Sweden; 4 Department of Medical Biochemistry and Cell Biology, University of Gothenburg, Gothenburg, Sweden; 5 Department of Medical Sciences, Clinical Bacteriology, University of Uppsala and Uppsala University Hospital, Uppsala, Sweden; University of Hyderabad, India

## Abstract

*Campylobacter jejuni* is a common cause of bacterial gastroenteritis in most parts of the world. The bacterium has a broad host range and has been isolated from many animals and environments. To investigate shedding patterns and putative effects on an avian host, we developed a colonization model in which a wild bird species, the European Robin *Erithacus rubecula*, was inoculated orally with *C. jejuni* from either a human patient or from another wild bird species, the Song Thrush *Turdus philomelos*. These two isolates were genetically distinct from each other and provoked very different host responses. The Song Thrush isolate colonized all challenged birds and colonization lasted 6.8 days on average. Birds infected with this isolate also showed a transient but significant decrease in body mass. The human isolate did not colonize the birds and could be detected only in the feces of the birds shortly after inoculation. European Robins infected with the wild bird isolate generated a specific antibody response to *C. jejuni* membrane proteins from the avian isolate, which also was cross-reactive to membrane proteins of the human isolate. In contrast, European Robins infected with the human isolate did not mount a significant response to bacterial membrane proteins from either of the two isolates. The difference in colonization ability could indicate host adaptations.

## Introduction

Infections caused by *Campylobacter jejuni* account for a large proportion of cases of human bacterial gastroenteritis in industrialized countries [1]. The *C. jejuni* bacterium has been isolated from a range of sources, including surface and ground waters [2], [3], domestic [2] and wild mammals [4], [5], insects [6]–[8], and wild birds [9]–[13]. The most prominent source for human infections is consumption of chicken meat, either directly or through cross-contamination with other food items [14]. Although it is a diverse multi-host pathogen, there are sets of *C. jejuni* genotypes that seem restricted to certain hosts, and some genotypes are associated more often with human disease than others [14]–[16]. The existence of these genotypic complexes despite frequent recombination suggests niche adaptations [14], [16], in which strain-specific colonization properties evolve as a response to different selection pressures in different hosts.

Based on a temperature optimum similar to the temperatures found naturally in avian intestines, and the high prevalence of infection in the poultry population, wild birds have been suggested as a reservoir for the bacterium. Indeed, wild birds frequently are colonized with *C. jejuni*, although prevalence rates vary between host species and studies. Certain taxa of wild birds seem to be colonized more frequently by *C. jejuni*, while others appear not to be colonized at all [9], [10]. Host diet and feeding modes seem to be especially important factors in explaining the prevalence of *Campylobacter* species among host taxa [9], [10], [17]. However, serotyping and genotyping of isolates have shown that the largest proportion of *C. jejuni* genotypes occurring in wild birds are restricted to these hosts, and that only a minority of genotypes are shared between human/poultry and wild birds [11]–[13], [17]–[20]. These findings suggest that certain strains of *C. jejuni* may have adapted to different wild bird hosts [11], [13], which is supported by the pattern seen among clinical and veterinary sources [14], [16], [21], [22], or to survival and transmission in particular environments.

Although important, descriptive screening studies are limited in the sets of questions they can answer. To better understand *C. jejuni* biology in wild birds, a number of fundamental questions need to be addressed. For instance, to elucidate the role of wild birds as reservoirs for *C. jejuni* or to identify host-specific strains, we must know how long wild birds are colonized and whether the duration and intensity of bacterial shedding vary depending on strain origin. Furthermore, does colonization of *C. jejuni* have any measurable effects on body condition, or are wild birds asymptomatic carriers similar to chickens? To answer such questions, we developed a wild bird colonization model using European Robins *Erithacus rubecula* and challenged this model with *C. jejuni* isolates from different origins.

We chose the European Robin as the model species because of its relative abundance in Europe, its medium size (which makes it suitable for small aviaries), and its diet. In a previous study, no *Campylobacter* spp. were isolated from this species of bird, even though nearly 300 individuals were sampled [10]. Since *Campylobacter* spp. frequently infect the ecologically and phylogenetically related *Turdidae* species [10], we investigated the reasons for this pattern. In this paper, we show that the European Robin can readily be colonized with a *C. jejuni* isolate obtained from a closely-related bird species and that this isolate causes a specific immune response, as well as measurable effects on body condition. In contrast, a human isolate did not colonize the European Robins. Our results indicate strain-specific differences in colonization ability.

## Materials and Methods

### Ethics Statement

Ethical approval for trapping, sampling, and keeping of birds was obtained from the Malmö/Lund Animal Research Ethics Board (M139-03).

### Bacterial Strains

We used two *C. jejuni* isolates for the infection experiment: 00-4:268 was isolated from a Song Thrush *Turdus philomelos* trapped at Ottenby Bird Observatory (56°12'N, 16°24'E) in SE Sweden on 20 October 2000 [10], and 00F4382 was isolated from a young human male with a domestically-acquired *C. jejuni* infection in adjacent Kalmar County in the summer of 2000. Both isolates were retrieved from fecal matter using routine culture methods for *Campylobacter* [10].

The 00-4:268 isolate was chosen from a collection of isolates that had been investigated by pulsed-field gel electrophoresis (PFGE) [19] and multilocus sequence typing (MSLT) [15]. The PFGE macrorestriction profile of this isolate was distinct from those of human patient isolates in our collection and was of a sequence type common among Song Thrushes in Sweden (the ST-1315 genotype, which groups in the ST-1304 clonal complex).

The human isolate had the ST-48 sequence type, the central genotype of the ST-48 clonal complex. The patient had suffered diarrhea, vomiting, and fever, and had recovered after 1 week without treatment. Both strains were stored at −70°C immediately upon isolation until the onset of the experiment, without any passage on plates or in liquid media. The strains were picked directly from the frozen stock cultures and subcultured on blood agar plates for 48 h before challenge of the birds.

Both isolates are available at the Culture Collection of the University of Gothenburg (CCUG) under the accession numbers CCUG 59141 (human isolate) and CCUG 59142 (Song Thrush isolate).

### Trapping, Pre-Experiment Treatments, and General Handling of Birds

Wild European Robins *Erithacus rubecula* were trapped during autumn migration in September 2003 at Ottenby Bird Observatory, during the normal ringing program of the observatory. The European Robin is a small passerine songbird (15–25 g) of the *Muscicapidae* family and is a common species in most forested areas over large parts of Europe. Trapped birds were banded, measured, and then individually caged in small aviaries in two well-ventilated rooms (the control group in one room and the two treatment groups in a separate room) under a simulated natural light regime.

Each bird was screened for *Campylobacter* spp. infections by culturing fresh fecal samples on *Campylobacter*-selective plates in a microaerobic environment (see description below). One bird excreted hippurate-hydrolysis-negative *Campylobacter* spp.; consequently, this bird was released and replaced by a *Campylobacter*-negative bird. Another three birds were replaced because they would not eat the provided food. Eventually, we had 24 juvenile European Robins habituated to the aviaries for at least 4 days that were repeatedly culture-negative for *Campylobacter* (all birds were negative for at least 3 consecutive days prior to the start of the experiment). The birds had constant access to Lesser Mealworm *Alphitobious diaperinus* larvae and water supplemented with mineral and vitamins throughout the study. Food, water, and paper covering the bottoms of the cages were changed daily.

### Design of the Colonization Model

The 24 birds were divided into three groups of eight birds each: a control group (C: birds A–H) and two treatment groups: treatment 1 (TR1: birds I–P) and treatment 2 (TR2: birds Q–Y). The starting day of the experiment was designated as day 0, and the experiment lasted until day 25 (Table 1).

**Table 1:** Experimental setup and outcome.

	Control group								Treatment group 1								Treatment group 2							
Day post-infection	A	B	C	D	E	F	G	H	I	J	K	L	M	N	O	P	Q	R	S	T	U	V	X	Y
0									#	#	#	#	#	#	#	#	*	*	*	*	*	*	*	*
1									M	M	S	R	S	M	M	M			S			S		S
2									M	M	S	M	M	S	M	S								
3									S	M	S	M	S		M	S								
4										S	S	M	S	S	S	S								
5											S	M			S									
6												S		S										
7										S		M			S									
8										S		S	S		S									
9										S		S			S									
10												S												
11																								
12																								
13																								
14																	*	*	*	*	*	*	*	*
15																				M				
16																								
17																								S
18																								
19	R^a^*	S*	R*	R*	R*	M*	M*	*																
20	R																							
21	S																							
22																								
23																								
24																								
25																								

a samples for the control birds at day 19 were taken 5 h post-infection the same day as the challenge occurred.

Columns A–Y refer to individual birds. *Campylobacter jejuni* growth for each day is denoted as follows: R for rich growth (>100 cfu), M for median growth (11–100 cfu), and S for sparse growth (1–10 cfu). The challenge day is indicated with * for the human 00F4382 *C. jejuni* isolate and # for the Song Thrush 00-4:268 isolate.

On day 0, group TR1 was challenged orally with a suspension of the Song Thrush isolate (00-4:268), while group TR2 was challenged orally with a suspension of the human isolate (00F4382). Each oral challenge consisted of an infective dose of 10^5^ colony forming units (cfu) of 48-h *C. jejuni* cultures suspended in 50 µL sterile water. On the same day, the control group was sham-challenged orally with sterile water.

The birds were followed until the last shedding bird was culture-negative for *C. jejuni* for a period of 3 days. Since a striking difference was observed in bacterial shedding between the TR1 and TR2 groups, we decided to re-challenge the TR2 birds with a similar suspension of the 00F4382 isolate at day 14 and to challenge the control group at day 19 with the 00F4382 isolate (Table 1).

### Measurements, Faecal Sampling, and Bacterial Isolation

The birds were handled only once each day, usually between 10:00 AM and 12:00 PM. On each of these occasions, the birds were taken out of their cages and their body mass (to the nearest 0.1 g) and fat score levels were recorded on a scale 0–6 [23] as an index of the birds' body condition. While each bird was measured, a sample of fresh fecal material was collected from the bottom of its cage. Each cage was then cleaned and the food and water changed. The fecal samples were collected with a sterile swab, immediately placed in a charcoaled transport medium (Transwab, BioDisc, Solna, Sweden), and refrigerated.

The samples were transported daily to our laboratory where they were cultivated on traditional *Campylobacter-*selective, blood-free medium (45.5 g/L *Campylobacter*-selective agar base LAB M/LAB 112, 2 ampoules cephoperazone/amphotericin supplement LAB M/X 112, Bury, England) at 42°C in a microaerobic atmosphere (85% N_2,_ 10% CO_2_, 5% O_2_). Plates were examined after 48 and 72 h, and the resulting colonies were inspected and counted. Growth was categorized as either rich (>100 cfu), medium (11–100 cfu), or sparse (1–10 cfu). At least one colony per plate was tested for reactions using catalase, oxidase, and hipurate-hydrolysis tests to confirm *C. jejuni*.

Isolates from the challenged birds were compared with the strains prior to infection by PFGE analysis. We used the *Sma*I and *Kpn*I restriction enzymes, according to a previously described protocol [19], and a CHEF apparatus model DR III for electrophoresis (Bio-Rad Laboratories, Sundbyberg, Sweden). Digital gel photographs of the digests were analyzed with the Molecular Analyst Software Fingerprinting Plus software (version 1.6, Bio-Rad Laboratories, Sundbyberg, Sweden) and compared visually for differences and similarities between isolates.

### Blood Sampling

Blood samples were taken from the birds on four occasions, day -1 (the day before the start of the experiment), day 6, day 13, and day 25 (the termination of the experiment). These small samples, consisting of <50 µL peripheral blood, were obtained through drainage of the brachial vein of the bird's left wing using heparinized blood collecting tubes. Samples were spun at 2000×*g* in a tabletop centrifuge for 3 min, after which the plasma was collected, transferred to new tubes, and frozen at −20°C.

### *C. jejuni* Outer Membrane Preparations

*C. jejuni* outer membranes were prepared according to established methods [24]. The two isolates were grown in a microaerobic environment on blood agar plates for 48 h at 42°C after which bacteria were harvested and suspended in PBS. The samples were centrifuged at 5,000×*g* for 10 min, washed once with PBS, and centrifuged again at 5,000×*g* for another 10 min. The resulting pellets were re-suspended in 30 ml of 10 mM Tris (pH 7.5) and sonicated four times for 30 s, with 30-s rest periods between sonications (Sonorex RK 100, Bandelin, Germany). The suspensions then were centrifuged twice at 5,000×*g* for 20 min to remove intact cells. The supernatants were centrifuged at 100,000×*g* for 2 h at 4°C. The pellets were resuspended in 3 ml sterile ddH_2_O and subsequently added to 20 ml of sodium lauryl sarcosinate in 7 mM EDTA and incubated for 20 min at 37°C. The suspensions were centrifuged at 100,000×*g* for 2 h, the supernatant removed, and the pellets dissolved in 10 mM Tris (pH 7.5) and centrifuged again at 100,000×*g* for 2 h. Finally, each pellet was dissolved in 1 ml of sterile ddH_2_O.

### ELISA

European Robin plasma samples were tested using three different enzyme-linked immunosorbent assays (ELISAs). In the first assay, we measured the total level of circulating immunoglobulins (see [25]). Briefly, ELISA plates were coated with 100 µl of anti-chicken IgG (donkey anti-chicken IgG, ICN Biomedicals, Cat. No. 67–645) at a concentration of 6 µg/ml suspended in carbonate buffer (0.15 M, pH 9.6). Plates were incubated overnight at 4°C. The next day plates were blocked for 2 h with 3% powdered milk diluted in PBS-Tween 20 at room temperature. The plasma samples were diluted 1∶75 in diluent (1% powdered milk, PBS-Tween 20). After washing, diluted plasma samples were added to the plate in duplicate. At least two blank wells (containing only diluent) were included on each plate. The plates were incubated a second time overnight at 4°C. On the third day, 100 µl of rabbit-anti-red-winged blackbird IgG (1∶1000 in diluent), previously demonstrated to detect antibodies in other passerine species [25], [26], was added to each well of the plates after washing. The plates were incubated for 1 h at 37°C. After a second wash, 100 µl of peroxidase-labeled goat-anti-rabbit serum antibody (Sigma-Aldrich, Sweden, cat. A-6154) diluted at 1∶2000 was added to each well and the plates incubated for another 45 min at 37°C. The plates were washed and 100 µl of peroxidase substrate (2,2-azino-bis-3-ethylbenzthiazoline-6-sulfonic acid, ABTS; Sigma cat. A1888) and peroxide were added to all wells. The plates were immediately transferred to a V_max_ (Molecular Dynamics) kinetics ELISA reader. The plates were read at 30-s intervals for 14 min using a 405-nanometer wavelength filter. All antibody concentrations are reported as the slope of the substrate conversion (in 10^−3^× optical densities (OD); m_OD_) over time (m_OD_min^−1^). We calculated the mean of the duplicate values for each sample to obtain an antibody titer value. The mean value of the blanks was subtracted from the measured antibody titer to account for non-specific binding. On each plate, we included a dilution series of a standard sample that covered the range of antibody titers for the European Robins. We used the differences between the standard curves to account for between-plate variation.

In the second and third assays, we measured the birds' specific antibody production to each *C. jejuni* isolate. In the second assay, ELISA plates were coated with 100 µl of outer membrane extracts of the 00-4:268 isolate at a concentration of 54 mg/ml, and in the third ELISA assay, plates were coated with 100 µl of outer membrane extracts of the 00F4382 isolate at a concentration of 54 mg/ml. The *C. jejuni* outer membrane extracts were suspended in carbonate buffer (0.15 M, pH 9.6), and the plates were incubated overnight at 4°C. The next day the plates were blocked for 2 h with 3% powdered milk diluted in PBS-Tween 20 at room temperature. Plasma samples from the European Robins were diluted 1∶200 in diluent (1% powdered milk, PBS-Tween 20). After washing, diluted plasma samples were added to the plate in duplicate. We also included at least two blank wells (containing only diluent) on each plate. The plates were incubated overnight at 4°C. Methods on the third day, and those for calculating antibody titers, were the same as those described above for quantifying total antibody titers.

### Bacterial Migration in Soft Agar

Swarming assay was performed to assess the ability of the *C. jejuni* isolates to migrate in soft agar. The strain 81–176 was used as a positive control, and the non-motile FlaA/FlaB negative mutant 81–176flaAB- as a negative control. All strains were cultured on blood agar plates in a microaerobic environment at 42°C for 24 h before inoculation on soft agar plates. Brucella medium (Difco, Detroit, USA) containing 0.4% agar (Agar NO 1, Oxoid, Basingstoke, UK) was poured into Petri dishes and allowed to cool. The three isolates, 81–176, 00F4382, and 00-4:268, were inoculated into one plate in triplicate, and the experiment was performed three times. The isolates were inoculated by pushing a filled inoculation loop into the agar. A new layer of tempered Brucella agar (45°C) was poured on top of the plates to prevent swarming on the agar surface. Plates were incubated at 42°C for 24 h.

### Invasion Efficiency Assay

The invasion efficiency of the isolates was tested by the gentamicin resistance assay (reviewed by [27]). Briefly, INT 407 cells were grown in EMEM supplemented with 2 mM of L-Glutamine, 1% non-essential amino acids, 10% fetal bovine serum, and Penicillin/Streptomycin, all purchased from the Swedish Veterinary Institute, Uppsala, Sweden, except FBS, which was purchased from Invitrogen. Two days before the experiment, cells were seeded into 24-well plates (Nunc A/S, Roskilde, Denmark). Bacteria were grown in Brucella broth for 16 h before the experiment, pelleted by centrifugation at 8000×*g* and re-suspended in infection medium (EMEM +1% FBS) to a concentration of 2×10^8^ cfu/ml. Before the experiment, cells were washed with PBS, and then inoculated with 0.5 ml of the bacterial suspension (1×10^8^ cfu) and incubated for 2 h at 37°C in 5% CO_2_. Cells were washed three times with PBS, and 1 ml of infection medium containing gentamicin 100 mM was added to each well before another 2-h incubation period. Cells were washed twice with PBS before they were lysed with Triton X-100 (0.1% in ddH_2_O) for 30 min at 37°C in 5% CO_2_. The lysate was serially diluted, and bacteria were enumerated by plate count on blood agar plates. The concentrations of the bacterial inoculates also were determined by plate counts, and the invasion efficiency was calculated as the percentage of the inoculate that infected the cells.

### Cytokine Release

The ability of the bacterial isolates to trigger a cytokine response in INT 407 cells was determined essentially as described by Hickey *et al.* [28]. Cells were maintained in EMEM supplemented with 2 mM of L-glutamine, 1% non-essential amino acids, 10% fetal bovine serum, and Penicillin/Streptomycin, and were seeded into 24-well plates as described above. Before the experiment, cells were washed with PBS, inoculated with 0.5 ml of bacteria (2×10^8^ cfu/ml) in infection medium or medium control, and incubated at 37°C in 5% CO_2_. After 24 h, the supernatants were collected and centrifuged at 300×*g* for 10 min to remove cell debris. The supernatants were transferred to new tubes and immediately frozen at −20°C until the IL-8 concentration was measured by ELISA (Human IL-8 single analyte ELISA kit, SA Biosciences, Frederick, MD, USA) according to the manufacturer's instructions.

## Results

### *C. jejuni*-Colonization

All birds in the TR1 group were colonized by the 00-4:268 Song Thrush isolate. Excretion was most intense (growth categories medium to rich) during the first 3 days after infection, but at least three birds excreted detectable *C. jejuni* bacteria at 9 days after infection, and one bird (L) showed excretion on day 10 (Table 1). The average duration of excretion was 6.8 days (SE 0.912) for this group.

TR2 birds were infected with the human 00F4382 isolate on day 0 and re-challenged with the same isolate at day 14. At first exposure, only three birds (S, V, and Y) excreted *C. jejuni* after infection. All cases were observed on day 1 after infection and growth was sparse (Table 1). The repeated challenge on day 14 resulted in two colonized birds (birds T and Y), of which one excreted bacteria only for 1 day after infection and the other with a single isolate at 3 days after re-infection (Table 1).

The European Robins in the control group were infected with patient 00F4382 isolate on day 19. *C. jejuni* was detected in 7 of 8 birds 5 h after infection, but only in one bird 24 h after infection (Table 1). Unfortunately, this bird (bird A) was injured during handling and had to be removed from the experiment, precluding estimation of its duration of shedding.

### Changes in Body Mass and Fat Levels

**Figure 1:**
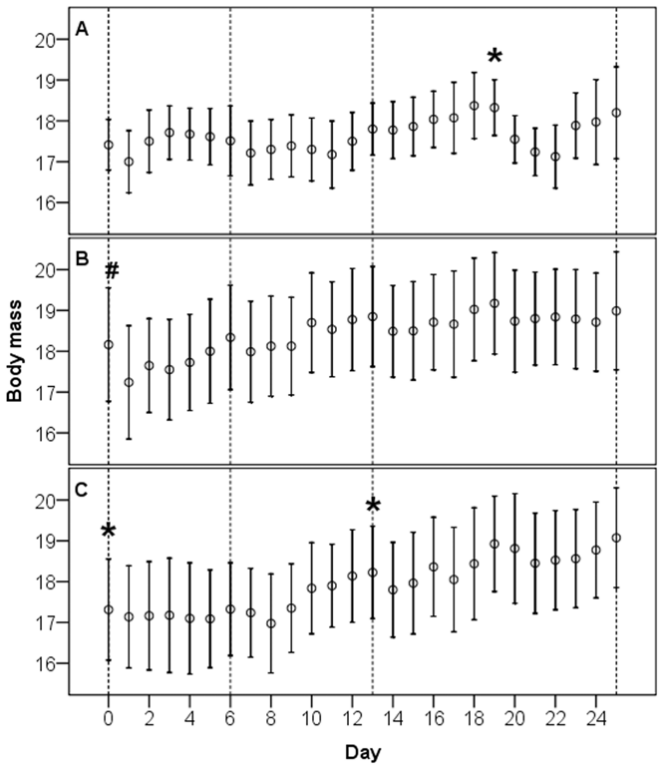
Body mass changes in European Robins *Erithacus rubecula* over the course of the infection experiment. Body mass is presented as the mean (±2 SE) of all individuals in each treatment group for each day over the course of the experiment. Challenge time points are marked with stars (*) for the human *C. jejuni* isolate 00F4382 and a bracket (#) for the Song Thrush isolate 00-4:268. Blood sampling time points are noted with hatched reference lines at days 0, 6, 13, and 25 post-infection.

We observed a general positive trend in body mass (Fig. 1) over the experimental period (Control: Pearson R = 0.166, p = 0.017, n = 208; TR1: R = 0.243, p<0.001, n = 208; TR2: R = 0.350, p<0.001, n = 208), with 20 birds showing significant positive trends in body mass and only one bird with a significant negative trend (bird B in the control group). Throughout the experiment, birds had significant fat stores, 2–6 on the scale used, and changes in mass also resulted in changes in fat score.

Blood sampling had a general negative effect on body mass. In cases where blood sampling was the only treatment (control bird blood samples 2–4, TR1 bird blood samples 2–4, and TR2 bird blood samples 2 and 4), birds lost 0.025–0.37 g from the day of blood sampling to the next day. These losses equalled relative body mass losses of 0.1–1.7%.

TR1 birds challenged with the Song Thrush isolate at day 0 had lost on average 0.93 g (SE 0.20; paired t-test, t = 4.95, d.f.  = 7, p = 0.002) at day 1 post-infection, equal to a 5.1% reduction in relative body mass. After day 1, average mass increased slowly and no significant effects on mass were seen at day 4 post-infection for these birds (Fig. 1; paired t-test, t = 1.98, d.f.  = 7, p = 0.09). TR2 birds did not show any changes in mass after the first challenge with the human isolate (day 0 vs day 1, paired t-test, t = 0.89, d.f.  = 7, p = 0.40), but were negatively affected by the second challenge (paired t-test, t = 7.20, d.f.  = 7, p<0.001) with average losses of 0.43 g (SE 0.06) between days 13 and 14. However, although significant, these losses were equivalent to only 2.4% of the relative body mass and were more in line with the maximum observed losses for birds bled but not challenged with *Campylobacter* (see above). The mass losses were more transient for the TR2 birds, with a tendency for birds to be leaner at day 15 (day 13 vs day 15, paired t-test, t = 2.35, d.f.  = 7, p = 0.051) but not at day 16 (day 13 vs day 16, paired t-test, t = −1.27, d.f.  = 7, p = 0.25; Fig. 1). Finally, the control birds showed remarkable declines in mass when challenged with the human strain at day 19 (Fig. 1). Initially, the birds in this group lost an average of 0.78 g (SE 0.12) between day 19 and day 20 (paired t-test, t = 6.59, d.f.  = 7, p<0.001), and this trend continued so that birds at day 22 had lost on average 1.20 g (SE 0.42), equal to 6.5% of their relative body mass. From day 23 and onwards, we observed no further effects on mass.

### Immune Response to *C. jejuni* Infection

**Figure 2:**
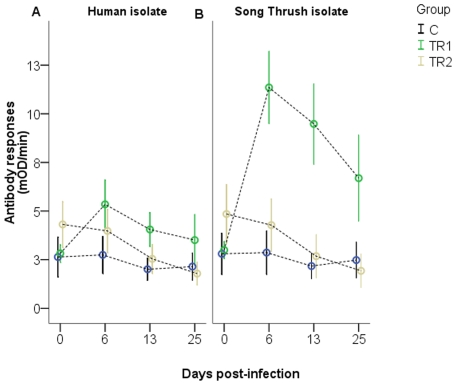
Development of antibody responses over the course of the infection experiment. For each treatment group and sampling occasion, the mean (with 95% confidence interval) of the birds' antibody responses (mOD/min) to membrane proteins of the (A) human *C. jejuni* isolate 00F4382 and (B) the Song Thrush isolate 00-4:268 are given. The control birds are shown in blue, TR1 birds in green, and TR2 birds in yellow. Samples were taken at days 0, 6, 13, and 25 post-infection.

Birds colonized with the Song Thrush isolate (TR1) produced high antibody titers against the outer membrane proteins of the infective strain. The response peaked 1 week post-infection (blood sample day 6), and then declined but remained elevated relative to the pre-infection response until the end of the experiment at day 25 (Fig. 2b). TR1 birds did not differ in response from the control group at day 0 (t = −0.38, df = 13, p = 0.71), but did at the remaining sampling time points (blood samples on days 6, 13, and 25, t = −3.98 to −9.32, df = 13–14, p<0.001). The TR1 group also showed an immune response to the human *C. jejuni* strain by ELISA with a curve similar, but less pronounced, to that seen against Song Thrush *C. jejuni* membrane proteins (Fig. 2a). This response was significantly stronger than in the control birds at days 6 and 13 after infection (t = −3.9 to −4.65, df = 14, p<0.02), whereas antibody titers were similar from TR1 and control birds at the beginning (day 1; t = −0.38, df = 13, p = 0.71) and the end (day 25; t = −2.10, df = 13, p = 0.56) of the experiment.

The TR2 group, challenged with human *C. jejuni*, had slightly higher antibody titers to the human strain at day 0 than the control group (t = −2.48, df = 15, p = 0.03) and the same pattern was observed for the titers against the Song Thrush *C. jejuni* strain (t = −2.51, df = 15, p = 0.02), suggesting that the TR2 group had somewhat higher levels of general antibodies against *Campylobacter* immediately before the experiment started. This difference could not be observed at later sampling occasions (t = 0.94 to −1.94, df = 14–15, p>0.07; Fig. 2a). The TR2 group did not show any significant antibody response to the Song Thrush *C. jejuni* isolate (t = 1.04 to −1.86, df = 14–15, p>0.08; Fig. 2b). Thus, despite two challenges with human *C. jejuni*, the birds in the TR2 group did not develop any significant increase in anti-*Campylobacter* antibodies.

Total Ig was not affected by infection with either isolate: neither TR1 nor TR2 showed significantly elevated total Ig levels compared to those of the control group (one-way ANOVA, F = 1.87 to −2.42, df = 2, p>0.11).

### Virulence of the Infecting Isolates

The virulence of the two isolates was assessed by three *in vitro* assays and compared to the virulence of *C. jejuni* strain 81–176. In the first test, the ability of the bacteria to migrate in soft agar was determined as a measure of motility. The migration distance in the agar differed significantly among the three tested isolates (Kruskal Wallis test, χ = 6.13, df = 2, p = 0.047), with the 81–176 reference strain migrating the shortest distance (23.7±1.9 mm), approximately 4–5 mm shorter than the other tested isolates (00F4382: 27.5±3.6 mm, 00-4:268: 28.2±2.2 mm). In the second test, we investigated the invasion efficiency of the isolates by a gentamicin resistance assay. The 00F4382 isolate had the highest invasion efficiency,with an average of 0.113% (±0.037) of the inoculated bacteria entering the INT407 cells. The Song Thrush isolate (00-4:268) had a lower invasion efficiency, 0.068% (±0.023), and the 81–176 strain was the least invasive, 0.015% (±0.006). Invasion efficiency was significantly different between the isolates (Kruskal Wallis test, χ = 6.97, df = 2, p = 0.030).

The third assay tested the ability of the isolates to trigger an IL-8 response in the intestinal epithelial cell line INT 407. Similar to the two tests above, the 00F4382 and the 00-4:268 isolates gave stronger responses than the 81–176 strain, in this case approximately 2–3 times more IL-8 after 24 h compared to 81–176 (00F4382 = 2392±78 pg/ml; 00-4:268 = 4041±306 pg/ml; 81–176 = 1312±252 pg/ml, medium control  = 371±82 pg/ml; Kruskal Wallis test, χ = 15.15, df = 2, p = 0.001).

## Discussion

Our experiment was developed to examine differences in colonization of *C. jejuni* isolates in a wild bird species, the European Robin. We found that these birds were readily colonized by oral challenge with the strain isolated from a closely-related bird species, the Song Thrush. This strain colonized all challenged individuals, and the birds excreted viable bacteria up to 10 days after infection. However, we observed a remarkable isolate-related difference in colonization ability as the three challenge attempts with the human-origin isolate resulted in only transient colonization in some birds (Table 1). The human isolate was viable, since samples collected 5 h after infection (instead of the normal 24-h lag-time) gave positive *C. jejuni* colonies from 7 of 8 tested birds (control birds at day 19).

The virulence of the two isolates was characterized in three different *in vitro* assays and compared to the virulence properties of the *C. jejuni* reference strain 81–176. The virulence of the 81–176 strain has been established in human subjects [29], and this strain is known to be highly invasive and to trigger strong cytokine responses in infected cells [28], [30]. Interestingly, both the human and the Song Thrush isolates migrated better than the 81–176 strain in soft agar, were more invasive in the gentamicin resistance assay, and triggered higher cytokine responses in INT407 cells. Invasiveness is a feature of virulent isolates of *C. jejuni* and has been shown to correlate with the cytokine response triggered in intestinal epithelial cells. In our study, the 81–176 strain triggered an IL-8 response that was comparable to that demonstrated by Hickey *et al*. [28] (554±156 pg/ml), when taking into account that we used half the inoculation volume. Together these results suggest that both infecting isolates can be considered virulent, and hence, that the lack of colonization of the European Robins with the human isolate cannot be attributed to impaired virulence or non-functional flagella.

It seems that the human strain passed quickly through the intestinal tracts of the birds and did not effectively colonize these avian hosts. Most studies in chickens have shown that both very young and older birds are susceptible to colonization, and that the dose needed for colonization may be several orders of magnitude lower than the dose that the European Robins in this study received [31]. Strain-related differences in colonization have been noted in chicken models [31], [32], as well as differences in susceptibility depending on chicken genotype [33]. Furthermore, the shedding period of European Robins colonized with the Song Thrush isolate was shorter than that seen normally in chickens colonized with *C. jejuni* which, once colonized, usually remain so until the time of slaughter.

In chickens, *C. jejuni* colonize the cecae and lower digestive tract (and sometimes also the spleen and the liver), normally without causing any symptoms, despite bacterial concentrations up to 10^10^ cfu/g in cecal contents [31]. In the European Robin model, we observed a strong humoral immune response against the colonizing Song Thrush isolate. The antibody titers peaked 1 week after infection and declined gradually thereafter, but remained elevated throughout the study period. These antibodies were cross-reactive in ELISA to the human *C. jejuni* isolate (Fig. 2a), although with lower affinity. In contrast, the birds infected with the human isolate (TR2 birds at day 0 and day 13, control birds at day 19) did not mount antibody responses (Figs. 2a and 2b), suggesting that colonization is required to evoke a humoral immune response in these birds. The induction of a specific antibody response was not mirrored in the total antibody levels, which did not show any statistical differences among the three groups.

Body mass and fat scores of the caged birds showed general positive trends over the course of the experiments (Fig. 1). Body mass increase is expected in European Robins at this time of the year, which coincides with autumn migration. European Robins, as well as most other migratory birds, use fat deposits as fuel for their migratory flights, and the trends in mass and fat scores measured in this study clearly show that this species was able to gain sufficient energy reserves while housed in cages. We did not expect the birds to develop gastroenteritis, given that the bacterium is common in many wild bird species [9]–[11] and in domestic poultry, and that disease manifestations are observed rarely among avian species infected with *C. jejuni* [31]. None of the birds in our study showed obvious signs of infection, such as lethargy, blood in the feces, or behavioral changes. However, significant mass losses were measured at certain intervals during the experiments. Blood sampling resulted in mass losses in the range of 0.1–1.7% of relative body mass, but these losses often were resolved within 1–2 days.

Colonization with the Song Thrush *C. jejuni* isolate resulted in significant weight loss in the infected birds (Fig. 1). This loss was on average 5.1% of relative body mass, which is more than twice that caused by maximum handling effects when sampled for blood alone. Compared to the control group, the birds needed (on average) a period of 4 days to regain their masses to pre-infection levels, and we interpret the observed mass loss of 2–3% as an actual response to the infection. The fat scores did not correlate with the rapid decreases in body mass in infected or blood sampled birds, implying that body mass loss was not simply due to utilization of stored fat.

TR2 birds infected with the human strain decreased marginally more in body mass than expected from the handling effect at blood sampling. However, when the control birds were infected with the human strain at day 19, they showed significant loss of mass (Fig. 1). Over the course of 3 days, the birds lost an average of 1.2 g, equal to 6.5% of their relative body mass. Although significant, these losses were not accompanied by prolonged shedding of *C. jejuni* or an elevated humoral immune response (Figs. 2a and 2b). One possible explanation for the increased mass loss is that this group of birds was handled again at 5 h after infection, which may have increased the handling-related effect on body mass.

The genetic differentiation between the two isolates varied between loci from 1.26% (6-bp difference) at the *asp*A locus to 4.73% (24-bp difference) at the *gly*A locus. *C. jejuni* is a highly diverse species and the level of sequence variation observed in this study is not unusual for typed *C. jejuni* isolates. The population structure of *C. jejuni* can be described as weakly clonal or epidemic [14], [15], where diversification of genotypes in clonal complexes occurs through point mutations and recombination events. The human isolate belonged to the ST-48 clonal complex, a set of genotypes frequently noted in human stool samples and in food animals, which has been isolated in more than 20 countries [14], [15]. This complex also is related to other widespread clonal complexes, such as the ST-21 complex [14]. Interestingly, a recent study conducted on wild birds in a free-range poultry farm environment in England found two wild European Starlings *Sturnus vulgaris* infected with *C. jejuni* of the ST-38 genotype, which falls within the ST-48 complex [13]. The Song Thrush isolate, on the other hand, belonged to a clonal complex of genotypes that seem restricted to thrushes. In our material, we have 13 isolates (eight from Song Thrushes and five from European Blackbirds *Turdus merula*) that belong to the ST-1304 complex. The other 76 *C. jejuni* Song Thrush isolates in our collection are distributed among clonal complexes with predominantly wild birds or the environment as identified sources (ST-677 complex, one isolate; ST-692 complex, one isolate; ST-1264 complex, 31 isolates; ST-1325 complex, one isolate; ST-1347 complex, 18 isolates; unassigned, 24 isolates). MLST studies have identified regional patterns in *C. jejuni* genotype distributions within poultry [34] and affinity between certain genotype assemblages and certain farm animal species [35]. However, the association between certain hosts and certain *C. jejuni* genotypes seem much stronger in wild bird species. For instance, both geese and European Starlings inhabiting the same free-range poultry farm environment in England carried markedly different populations of *C. jejuni*, and nearly all genotypes were restricted to wild birds [12], [13].

The European Robin is classified in the same family as thrushes of the genus *Turdus*, a group of birds that we earlier noted are frequently colonized by *C. jejuni* at this sampling locality [10]. However, although this bird is systematically related to the *Turdus* genus and has a diet that overlaps with thrushes, we had previously not isolated *Campylobacter* spp. from these birds, which led us to believe that they were rarely colonized by *C. jejuni* in nature. The results of the experiment show that European Robins are susceptible to colonization by *C. jejuni*, at least to *C. jejuni* bacterial strains that occur in related thrush species. Furthermore, when collecting birds for the experiment, we found one bird that excreted hippurate-hydrolysis-negative *Campylobacter*, indicating that infection also can occur in nature. European Robins and Song Thrushes have similar breeding ranges, migrate through the study area at approximately the same time and share a ground-foraging invertebrate feeding tactic. Our failure to detect *C. jejuni* in European Robins, while finding it in Song Thrushes and other thrushes [10] at the study site is intriguing. Since the European Robins were susceptible to infection, it may be that behavioral differences or differences in diet composition are the key. Furthermore, a recent study on wild starlings *Sturnus vulgaris* breeding in a farm environment revealed seasonal variation in *C. jejuni* strain diversity and in prevalence, as well as an effect of age, where nestlings and juveniles had a higher prevalence for infection than older birds [11]. On the other hand, the comparably short colonization period (average of 6.8 days) together with the specific immune response and weight loss in the birds could mean that these birds are generally naïve to colonization by *C. jejuni*. Further studies in a species known to carry *Campylobacter* spp. in nature, such as the Song Thrush or the European Starling, could better address these questions.

In conclusion, we developed a wild bird model for studying *C. jejuni* colonization. Like most other animal models [31], [36], the European Robin did not develop gastroenteritis, thus precluding its use as a model mimicking human disease. On the other hand, we successfully used this model for studying differences in the colonization abilities of different *C. jejuni* strains, showing that as a model, these birds could be used in the future to address questions regarding host specificity. Challenge with the human isolate did not produce more than transient colonization in the European Robins, while the Song Thrush isolate colonized the birds for 3-10 days, and at the same time, evoked a significant humoral immune response and a decrease in body mass. Although this study is limited, it indicates that European Robins are poor bacterial reservoirs for *C. jejuni* and that they likely are of little importance for human *C. jejuni* epidemiology. The results of the actual experiment as well as genetic analyses of wild bird *C. jejuni* datasets, which imply reduced genetic interchange between *C. jejuni* populations in different wild bird species [11], [13], [19], could, in our view, be interpreted as indications of host adaptations in *C. jejuni* that merit further study.
